# Anabolic steroids after total knee arthroplasty. A double blinded prospective pilot study

**DOI:** 10.1186/1749-799X-5-93

**Published:** 2010-12-15

**Authors:** Erik Hohmann, Kevin Tetsworth, Stefanie Hohmann, Adam L Bryant

**Affiliations:** 1 Musculoskeletal Research Unit, Central Queensland University, Australia, Department of Orthopaedic Surgery, Clinical Medical School, University of Queensland, Australia; 2 Royal Brisbane Hospital, Australia, Department of Orthopaedic Surgery, Medical School, University of Queensland, Australia; 3 Centre for Health, Exercise and Sports Medicine, University of Melbourne

## Abstract

**Background:**

Total knee arthroplasty is reported to improve the patient's quality of life and mobility. However loss of mobility and pain prior to surgery often results in disuse atrophy of muscle. As a consequence the baseline functional state prior to surgery may result in poorer outcome "post surgery" and extended rehabilitation may be required. The use of anabolic steroids for performance enhancement and to influence muscle mass is well established. The positive effects of such treatment on bone and muscle could therefore be beneficial in the rehabilitation of elderly patients. The purpose of this study was to investigate the effects of small doses of Nandrolone decanoate on recovery and muscle strength after total knee replacement and to establish the safety of this drug in multimorbid patients.

**Methods:**

This study was designed as a prospective double blind randomized investigation. Five patients (treatment group) with a mean age of 66.2 (58-72), average BMI of 30.76 (24.3-35.3) received 50 mg nandrolone decanoate intramuscular bi-weekly for 6 months. The control group (five patients; mean age 65.2, range 59-72; average BMI 31.7, range 21.2-35.2) was injected with saline solution. "Pre-operatively" and "post-operatively" (6 weeks, 3,6,9 and 12 months) all patients were assessed using the knee society score (KSS), isokinetic strength testing and functional tests (a sit-to-stand and timed walking tests). In addition, a bone density scan was used preoperatively and 6 month postoperatively to assess bone mineral density.

**Results:**

Whilst the steroid group generally performed better than the placebo group for all of the functional tests, ANOVA failed to reveal any significant differences. The steroid group demonstrated higher levels of quadriceps muscle strength across the postoperative period which reached significance at 3 (p = 0.02), 6 (p = 0.01), and 12 months (p = 0.02). There was a significant difference for the KSS at 6 weeks (p = 0.02), 6 (p = 0.02) and 12 month (p = 0.01). The steroid group demonstrated a reduction in the amount of bone mineral density at both the femur and lumbar spine from "pre-" to "post-surgery", however, these results did not reach significance (*p *< 0.05) using one-way ANOVA.

**Conclusions:**

This project strongly suggests that the use of anabolic steroids result in an improved outcome as assessed by the KSS and significantly increases extensor strength. No side effects were seen in either the study or control group.

**Trial Registration Number:**

Regional Health District: Register No. 03.05

Human Research Ethics Committee University: Clearance Number: 04/03-19

## Background

Osteoarthritis of the knee is one of the leading causes of pain and disability for the knee [1]. Total joint replacement is generally accepted as the main treatment for end-stage osteoarthritis. In fact it has revolutionized the treatment of disabling arthritis of the lower extremity [2]. Osteoarthritis of the knee is common and affects 10% of the population aged over 55 [3]. Close to 125.000 procedures were performed in the United States Medicare population [4] in 1995 and 20.000 were performed in Australia in 2009[5]. Long term studies on survivorship use end points such as revision surgery and reported survival rates between 84% and 98% at 15 years [6,7]. Whilst patients report an overall improvement after surgery the benefits after surgery are most significant for pain and stiffness 3 months after surgery [8]. Substantial functional improvement using effect sizes of outcome measures are higher rated by surgeons whereas patients derived measures showed effect smaller effect sizes [9]. Muscle strength, especially quadriceps strength has been shown to be highly correlated with functional performance and undergoes a decline after surgery [10,11]. Improving postoperative muscle strength could thus be important to accelerate recovery and enhance the potential benefits of total knee arthroplasty [10].

Anabolic steroids have long been used by athletes to improve their performance [12]. They have potent anabolic effects on the musculoskeletal system, including an increase in lean body mass, a dose-related hypertrophy of muscle fibers, and an increase in muscle strength and mass [13]. The use of anabolic steroids in elderly patients after knee replacement could therefore have beneficial effects on postoperative development of muscle strength. This possible may result in faster recovery and earlier mobilization. In addition anabolic steroids may have an effect on bone mineral density.

The purpose of this study was to investigate the effects of small doses of Nandrolone decanoate on recovery and muscle strength after total knee replacement. A research hypothesis was formulated that there would be a difference between the group who received anabolic steroids resulting in faster recovery, higher muscle strength and increased bone mineral density compared to the group that only received normal saline injections.

## Methods

Patients were recruited from the department of orthopaedic surgery outpatient clinics at a large regional academic teaching hospital. Prior to participation, all subjects were familiarized with the procedures and gave verbal and written informed consent in accordance with the Human Ethics Research Review Panel of the University and the Regional Health District. The study was designed as a prospective randomized double-blinded pilot project.

### Inclusion and Exclusion Criteria

Patients aged between 50 and 70 years and monolateral primary osteoarthritis were recruited. Those with rheumatoid arthritis were excluded to avoid the introduction of confounding variables. Patients where the administration of Nandrolone could result in severe side effects or in significant interaction with other drugs and possibly cause worsening of pre-existing conditions such as prostate hypertrophy were excluded. This also included: patients with cardiac conditions resulting in chronic ischaemia and acute coronary syndrome or an ejection fraction of less than 40%; patients with chronic liver disease and chronic renal failure; male patients with a symptomatic hypertrophic benign and malignant prostate, patients on antiepileptic medication such as Valproic Acid and Carbamazepine. All patients were routinely assessed by a specialist physician prior to enrolment. Recruitment continued until five patients in each group was achieved.

#### Randomization

Patients were allocated to either the steroid or control group by closed envelopes on the first day after surgery by the research coordinator. Randomization was carried out by a block of ten envelopes. The protocol was computer generated using an internet based generator http://www.randomization.com using 2 blocks of ten with 10 patients per block. This was done in order to guarantee continuation of randomization in case one of the patients needed to be excluded within the study period.

#### Surgical Protocol

All patients received a combination of regional and general anaesthesia. A standard dose of 2 g Cefazolin was administered prior to anaesthesia. All patients received a cemented total knee replacement (Stryker^® ^Duracon™) with a mobile bearing surface through a standard midline skin incision and parapatellar median approach. Surgery in all patients was performed by a single surgeon using a computer navigation system (Stryker^® ^Navigation) in all cases.

#### Postoperative Protocol

Postoperatively patients were admitted to the surgical ward. Cold compression with a Cryo/Cuff (Arthrex) was used intermittently for 24 hours in all patients. A continuous passive motion (CPM) machine was used from the first postoperative day. All patients were mobilized full weight bearing on day 1 post surgery. Intravenous antibiotics were continued for 24 hours and subcutaneous Enoxiparine (Clexane^®^) was commenced until discharge. As soon as patients were able to straight leg raise, flexion to 90 degrees actively was possible and a safe gait was achieved patients were discharged from the hospital. Sutures were removed routinely 12 days postoperative by their general practioner. Further follow up was performed by an independent examiner at the gait laboratory of the Musculoskeletal Research Unit of the University 6 weeks, 3, 6, 9 and 12 month following surgery. All subjects were also tested at this institution the week prior to surgery. The operating surgeon was only involved if the patient experienced significant side effects or complications either resulting from surgery such as infections, knee effusions or loss of motion. Patients were visited by the research nurse on day 2 or 3 after surgery whilst still hospitalized. Procedures were explained in detail and questions were answered. On day 5 patients received either 50 mg of Nandrolone decanoate or the equivalent volume of normal saline as an intramuscular injection. Patients were then visited every 2 weeks and injections with either normal saline or nandrolone was continued for a total of six months.

#### Outcome Measures

#### Functional Tests

##### Sit to Stand Test

A modified "sit to stand" and "timed walking test" as described by Bohannon [14] was performed" pre-operatively" and "post-surgery" as described earlier. Bohannon [14] measured the time (in seconds) subjects needed as they stood up and sat down from a firm padded armless chair of which the seat was 18.5 inches from the ground. We modified the protocol in consideration that elderly patients after total knee replacement would not be strong enough to repeatedly rise from a chair within 3 months after surgery. Patients were asked to stand up and sit down only once from a firm padded armless chair. Subjects were instructed to fold their arms across their chests before beginning the test. Subjects performed one timed trial. The stopwatch was started after the word "go" and stopped when the subject returned to the seated position.

##### Timed Walking Test

Speed of ambulation was assessed via electronic timing gates to record time to perform two laps between points 10 meters apart. A single set of gates was used. Subjects walked through the timing gates, to a marked position 10 meters from the start, pivoted and walked back. Total time to perform the task was recorded at two cadences. Initial cadence was at self-selected speed as described by Pollo et al [15] to familiarize and warm-up. Three trials were performed at maximal speed and average values were used for analysis. Subjects were then instructed to walk the same course at maximum speed. Reliability and responsiveness of this test has been demonstrated in healthy elderly populations [15,16].

##### Outcome Scores

The Knee Society Score (knee and function scores) was used in all cases. This rating system was introduced 1989 by Insall etal [17] and has become the standard evaluation system for reporting results after total knee replacement surgery. The KSS was found to have high intra- and interobserver variation [18,19] and reliable use necessitates evaluation by an experienced observer. However as this score is still the most commonly outcome system used and has adequate construct validity [19] we felt that the use of the rating system in combination with the other outcome measures would be sufficient to detect in between group differences.

##### Strength

Strength of the thigh musculature of the involved and non-involved limb of each subject was assessed using a Biodex™ Isokinetik Dynamometer. Quadriceps and hamstring concentric strength was determined at 180·s^-1^. Each subject performed one set of five maximal extension and flexion repetitions. On each test occasion the non-involved limb was tested before the involved limb. Peak torque generated by quadriceps and hamstring muscles were calculated from the three best trials. Peak torque was corrected for percentage bodyweight.

##### Bone Mineral Density

Bone mineral density (BMD) was measured with dual-energy xray absorptiometry (DEXA scan) using the LUNAR^® ^system. BMD was measured the week prior to surgery and repeated at six month following total knee replacement. DEXA was performed on the lower spine and neck of femur of the involved limb. The result was given in g/cm^2^. The results were not matched for age, weight, gender and ethnic origin as the influence of nandrolone on BMD over the six month interval was the measured variable.

#### Statistics

Means and standard deviations were calculated for age, height and mass and for the dependant variables derived from the functional assessment, quadriceps and hamstring muscle strength testing, knee society score evaluation and BMD assessment for the nandrolone and control groups. Independant samples t-tests were used to compare subject groups for age, height and mass and the knee society scores at pre-surgery, 3 months, 6 months, 9 months and 12 months. Similarly, an independant samples t-test was used to compare the BMD results at the spine and hip at pre-surgery and 12 months post-surgery. For the functional and the isokinetic tests a repeated measures ANOVA design was used to compare test limbs of the nandrolone and control groups across test occasions. Therefore, each ANOVA included two within factor (*test limb*: involved and non-involved and *test occasion*: pre-surgery, 3 months, 6 months, 9 months and 12 months) and one between factor (*subject group*: nandrolone and control). In the event of a significant (*p *< 0.05) main effect or interaction following ANOVA contrasts, *post hoc *comparisons of the means were conducted using the least significant difference (LSD) test to delineate differences amongst subject groups or between test limbs. Alpha level correction using Bonferroni or other such adjustments was not conducted so as to maintain statistical power. It is recognised that, whilst all the variables were carefully chosen, they are numerous and hence there is an increased risk of Type 1 error. However, the cost of incurring a Type 1 error was deemed minimal and therefore appropriate given the exploratory nature of the study. All analyses were conducted using Statistical Package for Social Sciences (SPSS, Version 12.0.1; Chicago, IL) for Windows.

## Results

### Subject characteristics

Ten patients were included in the study. The study group included 4 males and 1 female whilst the control group consisted of 3 males and 2 females. Descriptive data pertaining to the physical characteristics are presented in Table 1. Statistical analysis demonstrated no significant differences between subject groups for age, height, mass, or body mass index. Therefore, the two subject groups were considered to be appropriately matched on the main physical variables.

**Table 1:** Demographics of study and control group

	Study	Control
Height (cm)	173 (158-180)	167 (163-173)

Gender m/f	4/1	3/2

Weight (kg)	91 (71-105)	90 (56-110)

Age (years)	66.2 (58-72)	65.2 (59-72)

BMI (kg/m^2^)	30.8 (24.3-35.3)	31.7 (21.2-35.2)

### Knee Society Score

KSS and function scores (mean ± standard deviation) for the steroid and control groups are presented in Table 2 and 3. The KSS function scores improved across the post-operative period for both the nandrolone and control groups. Whilst there was a trend for the nandrolone group to demonstrate higher function scores, statistical analysis revealed no significant differences between subject groups. KSS revealed significant differences (p = 0.02-0.05) between subject groups post surgery except at 3 months where results just failed to reach significance levels (p = 0.07).

**Table 2:** Knee Society Score

	Pre-Op	6 wk	3 m	6 m	9 m	12 m
Study	54.6 (± 9.8)	80.4 (± 8.8)	85.4 (± 7.3)	90.6 (± 5.3)	90.8 (± 5.1)	91.4 (± 3.5)
Control	48.4 (± 2.3)	57.6 (± 10.2)	70.4 (± 9.4)	75.8 (± 11.0)	77 (± 10.6)	81.2 (± 7.1)
*p*-value	0.28	0.02	0.07	0.04	0.06	0.03

**Table 3:** Knee Society Function Score

	Pre-Op	6 wk	3 m	6 m	9 m	12 m
Study	55 (± 14.1)	56 (± 13.4)	66 (± 8.9)	78 (± 16.4)	84 (± 11.4)	88 (± 13.0)

Control	50 (± 0)	50 (± 0)	66 (± 15.2)	68 (± 13.0)	74 (± 11.4)	76 (± 16.6)

*p*-value	0.47	0.37	1.0	0.27	0.27	0.18

### Sit-to-stand

"Sit-to-stand" times (mean ± standard deviation) of the non-involved and involved limbs of the steroid and control groups are presented in Table 4. Statistical analysis revealed no significant main effects or interactions for the sit-to-stand data. There was, however, a near significant (p = 0.06) trend towards faster times for the steroid group at 9 months post-surgery compared with the control group.

**Table 4:** Sit to stand test (results in seconds)

	Pre - Op	6 wk	3 m	6 m	9 m	12 m
Study	9.9 (± 2.8)	8.8 (± 1.6)	7.4 (± 1.9)	8.3 (± 3.9)	6.7 (± 1.3)	7.4 (± 1.6)

Control	10.4 (± 6.0)	12.0 (± 5,4)	10.8 (± 4.8)	10.6 (± 6.2)	9.8 (± 2.9)	9.9 (± 2.2)

*p*-value	0.89	0.19	0.20	0.55	0.05	0.11

### Timed walk

Walking times (mean ± standard deviation) of the non-involved and involved limbs of the steroid and control groups are presented in Table 5. Like the results for the "sit-to-stand test", statistical analysis revealed no significant main effects or interactions for the walking data. Nevertheless, whilst the control group demonstrated only minor improvements throughout the testing cycle the nandrolone group improved steadily from 6 weeks to 6 months. At the 9 and 12 month intervals, however, the walking speed for the nandrolone group approximated towards the control group.

**Table 5:** Timed walk test (results in seconds)

	Pre-Op	6 wk	3 m	6 m	9 m	12 m
Study	21 (± 2.6)	23.3 (± 8.3)	18.4 (± 4.2)	17.9 (± 3.1)	18.9 (± 3.9)	21 (± 6.3)

Control	23.3 (± 2.4)	23.9 (± 1.4)	23.8 (± 5.1)	22.9 (± 4.9)	23.3 (± 3.6)	22.5 (± 3)

*p*-value	0.11	0.87	0.16	0.15	0.17	0.65

### Quadriceps and hamstring strength

Concentric quadriceps and hamstring isokinetic strength (mean ± standard deviation) for the non-involved and involved limbs of the steroid and control groups at 180·s^-1 ^are presented in Table 6 and 7. Statistical analysis of the quadriceps revealed significant between group differences at three (p = 0.02), six (p = 0.01), and 12 months (p = 0.02). No significant group differences by test interval interaction were observed for hamstring strength. Throughout the entire follow-up period the nandrolone group demonstrated steady improvement in both quadriceps and hamstring strength. In contrast the control group improved only minimal and did not reach pre-operative values for hamstring peak torque.

**Table 6:** Isokinetik Quadriceps Strength in Nm 180° sec1)

	Pre-Op	6 wk	3 m	6 m	9 m	12 m
Study	52.9 (± 14.1)	46 (± 11.0)	76.7 (± 21.9)	77.5 (± 24.4)	78.5 (± 32.2)	80.5 (± 34.9)
Control	49.7 (± 28.9)	39.6 (± 13.9)	47.1 (± 13.5)	55.1 (± 8.0)	63.1 (± 6.0)	55.8 (± 10.3)
*p*-value	0.78	0.59	0.02	0.01	0.30	0.02

**Table 7:** Isokinetic Hamstring Strength in Nm (concentric 180° sec1)

	Pre-Op	6 wk	3 m	6 m	9 m	12 m
Study	38.9 (± 22.5)	27.1 (± 14.7)	37 (± 18.5)	39.2 (± 12.2)	46.6 (± 26.4)	41.9 (± 23.9)
Control	25.7 (± 14.9)	12.9 (± 10.6)	15.9 (± 11.9)	21.7 (± 14.2)	27.1 (± 11.4)	23.9 (± 8.5)
*p*-value	0.19	0.22	0.1	0.11	0.18	0.15

### Bone Mineral Density

Bone mineral density (mean ± standard deviation) at the femur and spine for the steroid and control groups are presented in Table 8. None of the subjects demonstrated abnormal BMD values at any time point during the study. Bone mineral density at the femur and spine decreased from pre-surgery to 6 months post-surgery in both groups. However, the nandrolone group demonstrated a lower percentage change in BMD at both the femur and spine (femur: 0.71% versus 3.8%; spine: 1.25% versus 1.97%). Nevertheless, statistical analysis failed to identify any significant differences between subject groups.

**Table 8:** Bone Mineral Density (BMD) in g/cm2 at the femur and spine pre-op and 6 month postoperative

	Femur Pre-Op	Femur 6 m Post-Op	Percentage Bone Loss	Spine Pre-Op	Spine Post-Op	Percentage Bone Loss
Study	0.9246	0.918	-0.71%	1.358	1.341	-1.25%

Control	0.9337	0.898	-3.80%	1.27	1.245	-1.97%

*p*-value	0.9	0.82		0.19	0.06	

## Discussion

The results of this study indicate there are definite beneficial effects of Nandrolone for patients undergoing knee replacement surgery. The most obvious benefit is retention and significant improvement of quadriceps muscle strength as measured by isokinetic testing "pre-" and "post-operative".

Total knee replacement is a successful surgical procedure with clinical survivorship of 90 and 94% at 15 years [20] with a reported 85% patient satisfaction rate [21,22]. The outcome is associated with many factors. Marked functional limitations, a poor baseline status, low mental health scores and comorbidity are important pre-operative predictors [23,24]. Preoperative muscle strength has been identified to be one of the factors that influences functional outcome [25]. Patients with osteoarthritis have quadriceps weakness [22] which persists after surgery. Hsieh et al [26] demonstrated in patients with rheumatoid arthritis that minor joint involvement can cause muscle imbalance and joint instability. Berman et al [25] reported that patients with near normal quadriceps strength at minimum of 2 years after surgery had a more normal gait. Silva et al [27] measured isometric peak torque and found an average reduction of 30% of both extension and flexion peak torque. He could also demonstrate that relatively greater quadriceps strength was associated with a better functional score. Huang et al [28] reported that even after 6-13 years after surgery muscle balance still existed. Handel et al [29] compared a matched healthy group and found isokinetic muscle strength in patients 3 years after knee arthroplasty to be reduced by 30%. It may thus be important to address muscle weakness following surgery to improve outcome [30]. However there are only a few studies published assessing strength training after knee replacement. Rossi et al [31] investigated the effect of an 8-week resistive training protocol immediately after surgery and found torque production lower at 30 days post surgery compared to pre-operative levels but greater at 60 days. Thomas et al [32] used an isokinetic pulley system. Isokinetic strength increased to 90% to that of the unaffected knee within 16 days. Application of electric stimulation of the vastus medialis muscle resulted in a significant improvement in the patient's walking speed in a study by Avramidis et al [33].

Anabolic steroids have been used by athletes for half a century. Most of those athletes self administered high doses. Effects and side effects of those supraphysiologic doses are well documented in the literature [34]. Recently [13] there is an increasing interest in using anabolic steroids for medical conditions such as age related muscle wasting and increase muscle mass in patients with secondary wasting syndromes such as HIV. The main effects are positive anabolic actions on the musculoskeletal system influencing lean body mass, muscle size, strength, protein and bone metabolism and collagen synthesis [13]. The effect is dose dependent and significant increases in strength occur only with doses of 300 mg testosterone or more [13]. Side effects are rare and mostly benign and reversible [35].

The use of anabolic steroids may help to fasten the recovery of strength and mobility after total knee replacement. Our research has used 50 mg nandrolone decanoate intramuscularly biweekly which compared to testosterone has an enhanced anabolic and reduced androgenic effect. The safe use of this drug in frail elderly subjects has been demonstrated by Sloan et al [36]. In our study we have not observed side effects in any of our patients. However the drug was not self-administered but injected by an experienced research nurse. We could demonstrate that patients who received Nandrolone showed a clear trend towards better function as measured by the knee society functional score, functional tests, and a slower decrease of bone mineral density. Furthermore and more importantly we could demonstrate significant increases in isokinetic quadriceps peak torque, in the steroid group. This is even of more significant given the low numbers included in each group. The knee society score revealed significant differences after knee replacement between group subjects. In view of the non-significant differences in the functional tests, this may be due to the low numbers typical of a pilot project and should be viewed critically. However it was interesting to see that after cessation of Nandrolone the study group showed a trend to approximate to the control group which received normal saline injections. It could be argued that with the inclusion of more patients these effects could even be more significant. Even though none of those findings reach significance levels we have clearly demonstrated a positive effect of Nandrolone on postoperative recovery and a significant effect on strength development.

To our knowledge this is the only study investigating the effect of anabolic steroids after major joint surgery in a double-blind prospective fashion. Amory et al [37] has administered supraphysiological doses of testosterone enanthate (600 mg imi weekly) for 4 weeks to patients undergoing knee replacement. He noted a trend towards improvements in walking and stair climbing during the postoperative inpatient period. Hedstrom et al [38] treated women with hip fractures with a combination of 25 mg nandrolone every third week, vitamin D and calcium for twelve months and compared it to a control group receiving only Calcium. He showed that the nandrolone group despite the application of very low doses had a significantly higher Harris hip score, faster gait and demonstrated less bone loss and no loss of muscle volume measured by quantitative CT. A decrease in muscle strength mostly pronounced for the fast twitch type II fibre is a physiological fact [39,40] and may partially contribute to slower recovery after major surgery. The application of Nandrolone may thus only partially compensate for age related changes.

Possible limitations of this study include the introduction of selection bias. We were possibly unable to select a true random sample of subjects undergoing knee arthroplasty. Selecting from a highly motivated subgroup may have somehow lead to better outcome in both groups compared to the normal population. However the double-blind design minimized systemic error and eliminated observer and experimenter's bias. Due to the small number of subjects in each group measurement error can not be entirely excluded. Random errors and placebo effects however have most likely been eliminated as those effects would have appeared in both groups not substantially influencing results.

## Conclusions

The results of this research strongly suggest that nandrolone results in an improved clinical outcome as assessed by the knee society score and significantly increases quadriceps muscle strength after knee replacement surgery. A larger study is needed to confirm findings of this pilot project in order to recommend the general use of low dose anabolic steroids after joint replacement surgery.

## Competing interests

The authors declare that they have no competing interests.

## Authors' contributions

EH was the chief investigator, developed design and methods, analysed the data, drafted the manuscript and is responsible for the final approval of the manuscript. KT assisted with the design and analysis, assisted with the first draft and critically reviewed further versions. SH was the coordinator of the project; the only person who collected all data and injected subjects. She made substantial contributions to analysis and interpretation of the collected data. AB assisted with the design and analysis, assisted with the first draft and critically reviewed further versions. He also applied all statistical analysis and was involved in the interpretation of the results. All authors have read and approved the final manuscript
